# Prognostic impact of SCAI shock severity classes in AMI‐related cardiogenic shock: A sub‐study of the ECLS‐SHOCK Trial

**DOI:** 10.1002/ehf2.15446

**Published:** 2025-10-13

**Authors:** Janine Pöss, Jacob Jentzer, Steffen Desch, Hans‐Josef Feistritzer, Anne Freund, Michelle Roßberg, Christian Jung, Taoufik Ouarrak, Steffen Schneider, Ibrahim Akin, Tienush Rassaf, Tharusan Thevathasan, Uwe Zeymer, Holger Thiele

**Affiliations:** ^1^ Heart Center Leipzig Leipzig University and Leipzig Heart Science Leipzig Germany; ^2^ Department of Cardiovascular Medicine Mayo Clinic Rochester Minnesota USA; ^3^ University Hospital Düsseldorf Düsseldorf Germany; ^4^ Institut für Herzinfarktforschung Ludwigshafen Germany; ^5^ University Clinic Mannheim Mannheim Germany; ^6^ West German Heart and Vascular Center University Hospital Essen Essen Germany; ^7^ DZHK (German Center for Cardiovascular Research) Berlin Germany; ^8^ Deutsches Herzzentrum der Charité (DHZC), Campus Benjamin Franklin Berlin Germany; ^9^ Berlin Institute of Health Berlin Germany; ^10^ Department of Cardiology and Angiology, University Heart Center Freiburg‐Bad Krozingen, Faculty of Medicine University of Freiburg Freiburg Germany

**Keywords:** acute myocardial infarction, cardiogenic shock, extracorporeal life support, Society for Cardiovascular Angiography and Interventions

## Abstract

**Aims:**

The Society for Cardiovascular Angiography and Interventions (SCAI) Classification provides risk stratification of patients with acute myocardial infarction complicated by cardiogenic shock (AMI‐CS). This sub‐study of the ECLS‐SHOCK trial investigates the prognostic impact of SCAI stages in AMI‐CS and the influence of SCAI stages on the effect of extracorporeal life support (ECLS) therapy in AMI‐CS patients.

**Methods:**

Patients with AMI‐CS enrolled in the multicentre, randomized ECLS‐SHOCK trial were included. The outcomes, treatment effect and safety of ECLS were stratified according to SCAI stage at admission using a post‐hoc classification.

**Results:**

From a total of 417 patients enrolled in the ECLS‐SHOCK trial between June 2019 and November 2022, 51.6% (*n* = 215), 13.4% (*n* = 56) and 35.0% (*n* = 146) presented in SCAI Stages C, D and E, respectively. SCAI stages were associated with the risk of 30 day all‐cause mortality (C vs. D vs. E: 32.6% vs. 67.9% vs. 64.4%, *P* < 0.001), with rates of renal replacement therapy at 30 days (C vs. D vs. E: 7.0% vs. 19.6% vs. 13.7%, *P* = 0.03) and with poor neurological outcomes (C vs. D vs. E: 17.2% vs. 44.4% vs. 36.5%, P < 0.001). No interaction was observed between SCAI stage and the treatment effect of ELCS on 30 day all‐cause mortality (ELCS vs. control SCAI C: 32.7% vs. 32.4%; SCAI D: 68.4% vs. 66.7%; SCAI E: 59.7% vs. 68.4%, *P* for interaction = 0.65).

**Conclusions:**

In AMI‐CS patients included in the ECLS‐SHOCK trial, SCAI stages at admission were predictive for mortality and for the incidence of safety events. The efficacy of ECLS treatment was not affected by SCAI stage.

## Graphical abstract

Patients with AMI‐CS (lactate >3 mmol/L) enrolled in the multicentre, randomized ECLS‐SHOCK trial were included. From a total of 417 patients, 51.6% (*n* = 215), 13.4% (*n* = 56) and 35.0% (*n* = 146) presented in SCAI Stages C, D and E, respectively. SCAI stages were associated with the risk of 30 day all‐cause mortality, with rates of renal replacement therapy at 30 days and with poor neurological outcomes. No interaction was observed between SCAI stage and the treatment effect of ELCS on 30 day all‐cause mortality.

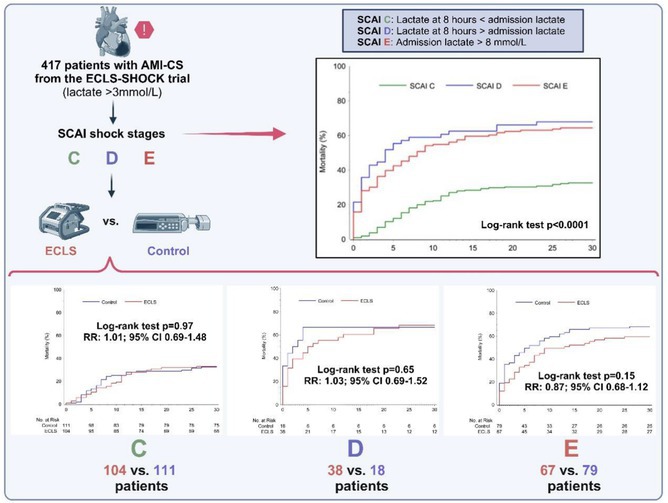

## Introduction

Cardiogenic shock (CS) caused by acute myocardial infarction (AMI‐CS) remains associated with short‐term mortality of up to 50% despite contemporary therapy including early culprit‐vessel revascularization.^1^, ^2^ Recent advances in temporary mechanical circulatory support (MCS) device technology have not demonstrated improved survival in randomized clinical trials (RCT) with routine use of MCS devices in unselected AMI‐CS cohorts.^3^, ^4^, ^5^ However, in the DanGer‐Shock trial performed in highly selected AMI‐CS patients without the risk of hypoxic brain injury, the use of a micro‐axial flow pump improved 6 month survival.^6^ This emphasizes the importance of disentangling heterogeneity within the AMI‐CS population to identify which patients may benefit from MCS.

The severity of AMI‐CS can vary widely between patients, and shock severity might be a plausible predictor of the potential benefit of temporary MCS.^7^, ^8^ The Society for Cardiovascular Angiography and Intervention (SCAI) shock classification is an established approach in determining the severity of AMI‐CS.^9^ Despite an array of different assessment strategies, most retrospective studies have emphasized that the SCAI shock stage on admission is incrementally associated with mortality in patients with AMI‐CS.^10^, ^11^, ^12^, ^13^, ^14^, ^15^, ^16^, ^17^, ^18^, ^19^, ^20^ Patients with higher SCAI shock stages may be considered for temporary MCS, but the effects of this strategy on survival are unknown.

ECLS‐SHOCK is the largest RCT of veno‐arterial extracorporeal membrane oxygenation, also called extracorporeal life support (ECLS), in patients with AMI‐CS and planned early revascularization.^3^ It showed no prognostic impact of ECLS with regard to the primary endpoint all‐cause mortality at 30 days compared with medical therapy alone. However, enrolled patients had substantial heterogeneity in shock severity. Accordingly, we sought^1^ to investigate the association of SCAI stage at admission with mortality, and^2^ to evaluate whether the SCAI shock classification influenced the safety and efficacy of ECLS in patients with AMI‐CS enrolled in the ECLS‐SHOCK trial population in more detail.

## Methods

### Study design, inclusion and exclusion criteria

The ECLS‐SHOCK trial was an investigator‐initiated, randomized, multicentre, open‐label trial investigating the prognostic impact of ECLS in addition to usual medical treatment in patients with AMI‐CS in whom early revascularization was planned as compared with medical treatment alone.^3^ The study protocol was previously published and was approved by the ethics committee at each participating site.^21^ In brief, patients with AMI‐CS (i.e., Stages C, D or E according to the SCAI classification)^9^ between 18 and 80 years of age were included in whom early revascularization was planned, preferably by percutaneous coronary intervention (PCI) or by coronary artery bypass grafting. CS was defined as a systolic blood pressure of <90 mmHg for >30 min or the initiation of catecholamines to maintain a systolic pressure of >90 mmHg, an arterial lactate level of >3 mmol/L, and signs of impaired organ perfusion with at least one of the following criteria: altered mental status, cold or clammy skin and limbs, or urine output of less than 30 mL/h. Patients with cardiopulmonary resuscitation (CPR) >45 min, mechanical cause of CS and patients being ineligible for ECLS insertion related to severe peripheral artery disease were excluded. Patients were randomized in a 1:1 fashion to ECLS in addition to usual medical treatment versus medical treatment alone immediately after coronary angiography. In patients randomized to ECLS, ECLS insertion was preferably performed prior to PCI.

### Risk stratification

For the present retrospective study, risk stratification was performed according to the SCAI stage at admission. Notably, the SCAI shock stages were not yet available when the ECLS‐SHOCK trial began enrolment. Therefore, a modified *post hoc* assignment of SCAI stages was used as described in the main ECLS‐SHOCK publication^3^: Stage C, a lactate level of 3–8 mmol/L at baseline with a lower‐than‐baseline lactate level at 8 h; Stage D, a lactate level of 3–8 mmol/L at baseline with a higher‐than‐baseline lactate level at 8 h or death within 8 h; and Stage E, a lactate level of >8 mmol/L at baseline. First, the impact of SCAI shock stages at admission on the primary and secondary endpoints as well as on safety outcomes was investigated within the entire study cohort of patients with AMI‐CS. Subsequently, the prognostic impact of ECLS was investigated according to the SCAI stage at admission.

### Study endpoints

The primary endpoint was all‐cause mortality at 30 days. Secondary endpoints included the need for renal replacement therapy, repeat revascularization, recurrent myocardial infarction, rehospitalization for congestive heart failure and poor neurological outcome defined as a Cerebral Performance Category (CPC) of 3 (severe neurologic disability) or 4 (persistent vegetative state) at 30 days. The safety endpoints were defined as moderate or severe bleeding defined as Types 3–5 according to the Bleeding Academic Research Consortium (BARC) criteria, stroke or systemic embolization and peripheral ischaemic vascular complications requiring surgical or interventional therapy.

### Statistical methods

All statistical analyses were performed according to the intention‐to‐treat principle. Continuous data are presented as median and interquartile range (IQR) or mean with standard deviation (SD) depending on the distribution of the data and were compared using the Mann–Whitney–Wilcoxon test. Categorical data and outcome‐related data are presented as absolute and relative frequencies and were compared using the *χ* ^2^ test or the Fisher's exact test, as appropriate. Additionally, risk ratios (RRs) with 95% confidence intervals (CIs) were estimated for outcomes. Kaplan–Meier analyses were performed comparing the outcomes of patients according to SCAI stage at admission, and for the effect of ECLS in addition to usual medical treatment compared with medical therapy stratified by SCAI stages, with groups compared using the log‐rank test. Results of all statistical tests were considered significant for *P* < 0.05. SAS software, version 9.4 (SAS Institute, Cary, NC, USA) was used for statistical analyses.

## Results

### Study population

**Figure 1:**
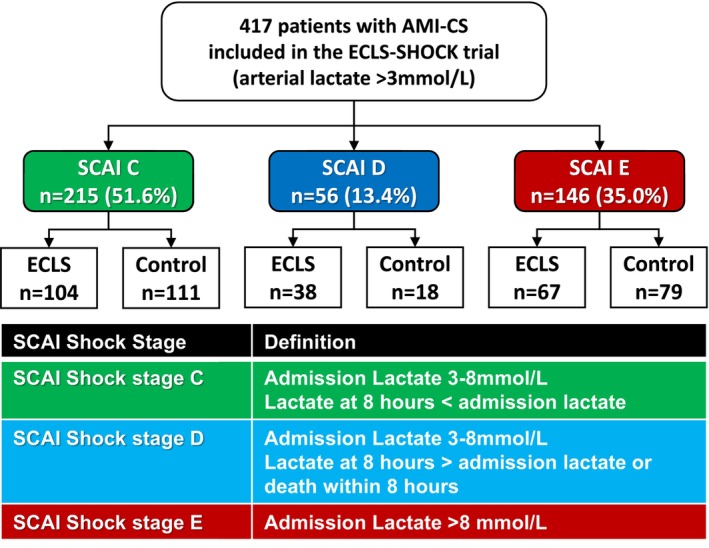
Study flow chart including distribution of SCAI shock stages. In the ECLS‐SHOCK trial, a total of 417 patients with an admission lactate level >3 mmol/L were included. Among them, 215 patients were classified as SCAI shock Stage C, 56 as Stage D and 146 as Stage E. AMI‐CS, acute myocardial infarction complicated by cardiogenic shock; SCAI, Society for Cardiovascular Angiography and Interventions.

From a total of 417 patients enrolled in the ECLS‐SHOCK trial between June 2019 and November 2022, 51.6% (*n* = 215), 13.4% (*n* = 56) and 35.0% (*n* = 146) presented in SCAI Stages C, D and E, respectively, *Figure* 1. Baseline characteristics according to SCAI stage at admission are provided in *Table* 1. No relevant differences were observed regarding age, and most patients were male in all groups (*P* = 0.50). The distribution of cardiovascular risk factors and prior medical history did not significantly differ comparing patients with different SCAI stages. Median lactate levels before PCI in SCAI Stages C, D and E were 5.1 versus 5.1 versus 11.1 mmol/L, respectively (*P* < 0.001). In line with this, pH was different between groups (C vs. D vs. E: median 7.2 vs. 7.2 vs. 7.1, *P* < 0.001). Differences between SCAI stages were observed regarding the prevalence of oliguria (C vs. D vs. E: 67.9% vs. 73.2% vs. 77.4%, *P* = 0.048) and admission serum creatinine levels (C vs. D vs. E: median 1.2 vs. 1.3 vs. 1.4 mg/dL, *P* < 0.001). Resuscitation rates (C vs. D vs. E: 75.1% vs. 70.4% vs. 84.2%, *P* = 0.052) and the duration of longest continuous cardiopulmonary resuscitation differed between groups. Moreover, differences were observed regarding the rates of epinephrine (C vs. D vs. E: 24.6%, 40.4% and 43.2%, *P* < 0.001) and dobutamine use (C vs. D vs. E: 29.5%, 48.1% and 43.9%, *P* = 0.004). Important angiographic findings and interventional treatment aspects did not significantly differ between SCAI stages. In patients undergoing ECLS, the time from ECLS insertion to support was similar, although the duration of ECLS treatment differed between groups (C vs. D vs. E: 2.3, 2.0 and 3.6 days, *P* = 0.023).

**Table 1:** Baseline/treatment characteristics and endpoints at ≤30 days according to SCAI stage.

	SCAI C	SCAI D	SCAI E	*P* value
Number of patients	215 (51.6%)	56 (13.4%)	146 (35.0%)	
Age in years	62.3 ± 9.3, *N* = 215	65.4 ± 9.3, *N* = 56	62.4 ± 9.9, *N* = 146	0.82
Male sex	82.3% (177/215)	82.1% (46/56)	79.5% (116/146)	0.60
Medical history				
Previous myocardial infarction	13.6% (29/214)	18.2% (10/55)	10.3% (15/145)	0.43
Previous stroke	7.9% (17/215)	9.1% (5/55)	6.2% (9/145)	0.57
Known peripheral artery disease	7.0% (15/214)	9.1% (5/55)	11.7% (17/145)	0.12
Atrial fibrillation	7.9% (17/214)	10.9% (6/55)	9.7% (14/145)	0.55
Impaired renal function	8.8% (19/215)	14.5% (8/55)	7.6% (11/145)	0.77
Signs of impaired organ perfusion				
Altered mental status	94.4% (203/215)	96.4% (54/56)	96.6% (141/146)	0.32
Cold, clammy skin and extremities	95.8% (206/215)	100.0% (56/56)	98.6% (144/146)	0.08
Oliguria with urine output <30 mL/h	67.9% (146/215)	73.2% (41/56)	77.4% (113/146)	0.05
Haemodynamic measurements at start of angiography				
Systolic blood pressure [mmHg]	100 ± 28, *N* = 182	106 ± 32, *N* = 45	102 ± 32, *N* = 110	0.53
Mean blood pressure [mmHg]	75 ± 21, *N* = 178	80 ± 26, *N* = 44	75 ± 20, *N* = 107	0.29
Heart rate [bpm]	91 ± 27, *N* = 176	98 ± 24, *N* = 44	93 ± 28, *N* = 110	0.67
Electrocardiographic rhythm prior to PCI				
ST‐segment elevation	68.9% (146/212)	51.8% (29/56)	70.6% (101/143)	0.88
Resuscitation within 24 h before randomization	75.3% (162/215)	69.6% (39/56)	84.2% (123/146)	0.06
Number of diseased coronary vessels				0.12
Single‐vessel disease	37.9% (80/211)	20.8% (11/53)	30.9% (43/139)	
Double‐vessel disease	29.4% (62/211)	34.0% (18/53)	31.7% (44/139)	
Triple‐vessel disease	32.7% (69/211)	45.3% (24/53)	37.4% (52/139)	
Coronary artery with culprit lesion				
RCA	25.1% (53/211)	15.1% (8/53)	28.1% (39/139)	0.63
LM	10.0% (21/211)	13.2% (7/53)	8.6% (12/139)	0.74
LAD	46.4% (98/211)	52.8% (28/53)	47.5% (66/139)	0.80
LCX	18.5% (39/211)	18.9% (10/53)	15.8% (22/139)	0.54
Admission laboratory values				
pH	7.2 ± 0.1, *N* = 177	7.2 ± 0.4, *N* = 43	7.1 ± 0.2, *N* = 122	<0.001
Lactate [mmol/L]	5.3 ± 1.5, *N* = 210	5.3 ± 1.5, *N* = 56	11.9 ± 3.1, *N* = 146	<0.001
Creatinine [mg/dL]	1.3 ± 0.6, *N* = 203	1.6 ± 1.5, *N* = 51	1.5 ± 0.7, *N* = 140	<0.001
Initial access site				0.47
Femoral	71.2% (153/215)	78.6% (44/56)	74.3% (107/144)	
Radial	28.8% (62/215)	21.4% (12/56)	25.7% (37/144)	
TIMI flow after revascularization				0.35
TIMI flow III	93.1% (188/202)	94.0% (47/50)	89.9% (116/129)	
ICU treatment				
Time between ECLS insertion and support [min]; Median (IQR)	10.0 (3.0, 18.0)	9.0 (1.0, 35.0)	11.0 (1.0, 24.0)	0.99
Days between ECLS start support and first removal; Median (IQR)	2.3 (1.6, 4.0)	2.0 (0.6, 4.5)	3.6 (1.8, 6.6)	0.023
Mechanical ventilation	91.0% (192/211)	88.9% (48/54)	94.3% (132/140)	0.30
Catecholamine requirement	96.3% (207/215)	92.9% (52/56)	95.2% (139/146)	0.58
Epinephrine	24.6% (51/207)	40.4% (21/52)	43.2% (60/139)	<0.001
Norepinephrine	91.3% (189/207)	90.4% (47/52)	86.3% (120/139)	0.15
Dobutamine	29.5% (61/207)	48.1% (25/52)	43.9% (61/139)	0.004
Duration of catecholamines [days]	7.7 ± 27.4, *N* = 181	6.4 ± 7.3, *N* = 202	6.3 ± 7.9, *N* = 132	0.34
Primary endpoint				
Death at 30 days	32.6% (70/215)	67.9% (38/56)	64.4% (94/146)	<0.001
Secondary endpoints at 30 days				
Renal replacement therapy	7.0% (15/215)	19.6% (11/56)	13.7% (20/146)	0.03
Repeat revascularization (PCI and/or CABG)	9.8% (21/215)	8.9% (5/56)	9.6% (14/146)	0.97
Poor neurological outcome (CPC 3–5)	17.2% (25/145)	44.4% (8/18)	36.5% (19/52)	0.002
Safety endpoints				
Peripheral ischaemic vascular complication	5.6% (12/215)	10.7% (6/56)	8.9% (13/146)	0.20
Stroke	1.4% (3/215)	3.6% (2/56)	5.5% (8/146)	0.03
All bleeding	17.2% (37/215)	26.8% (15/56)	23.3% (34/146)	0.12
Moderate or severe bleeding (BARC ≥3)	12.1% (26/215)	23.2% (13/56)	20.5% (30/146)	0.02

*Note*: Displayed are percentages and numbers or mean and standard deviation. *P* values: Pearson *χ* ^2^ test or Mann–Whitney–Wilcoxon test. Bold type indicates statistical significance.

Abbreviations: BARC, Bleeding Academic Research Consortium; CABG, coronary artery bypass grafting; CPC, cerebral performance category; ECLS, extracorporeal life support; LAD, left anterior descending; LCX, left circumflex; LM, left main; LVEF, left ventricular ejection fraction; *N*, number of observations; PCI, percutaneous coronary intervention; RCA, right coronary artery; SCAI, Society for Cardiovascular Angiography and Interventions; TIMI flow, Thrombolysis in Myocardial Infarction flow grade.

### Prognostic impact of SCAI stages at admission

**Figure 2:**
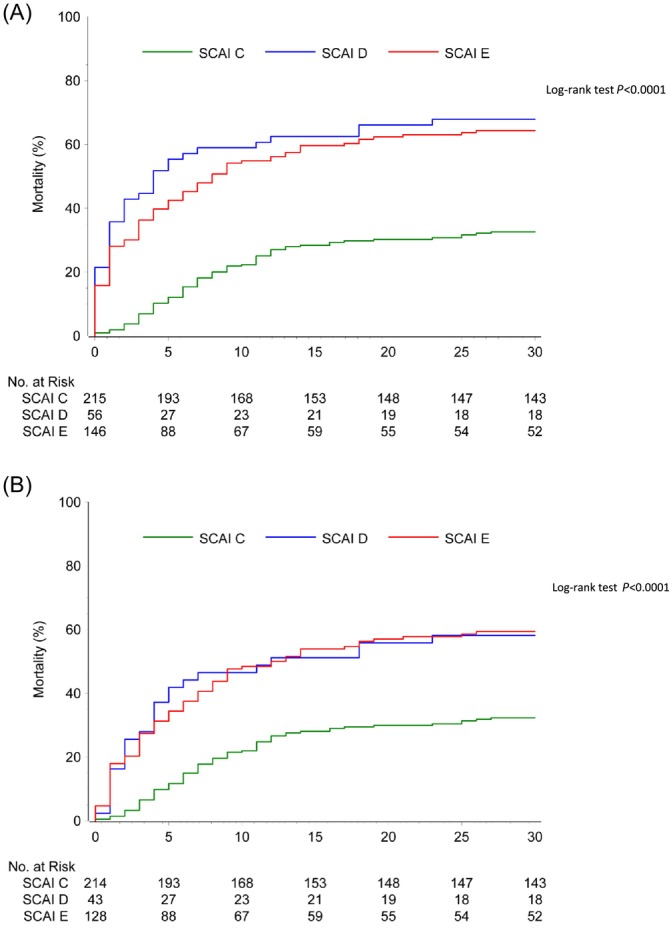
(A) Kaplan–Meier analysis investigating the prognostic impact of SCAI stages at admission. The numerically increased mortality of SCAI D compared with SCAI E patients can be explained by the post‐hoc SCAI classification of the ECLS‐SHOCK publication,^3^ which was used for this analysis. This classification included patients with lactate levels of <3–8 mmol/L at baseline with a higher‐than‐baseline lactate level or death at 8 h in the SCAI D group, thereby increasing the odds for inclusion of early deaths. (B) Kaplan–Meier analysis investigating the prognostic impact of SCAI stages at admission after exclusion of early deaths (within 8 h) in order to reduce the bias described in legend of *Figure* 2A. SCAI shock Stages D and E showed higher mortality rates compared with Stage C in both analyses, regardless of whether patients with early death were included or excluded (log‐rank *P* < 0.0001 for both). SCAI, Society for Cardiovascular Angiography and Interventions.

Main endpoints according to SCAI stage are depicted in *Table* 1. At 30 days, the primary endpoint all‐cause mortality occurred in 32.6% versus 67.9% versus 64.4% of patients admitted in SCAI Stages C, D and E, respectively (*P* < 0.001). Accordingly, SCAI stages at admission were associated with the risk of 30 day all‐cause mortality (SCAI D vs. SCAI C: RR = 2.01; 95% CI 1.60–2.71; SCAI E vs. SCAI C: RR = 1.98; 95% CI 1.58–2.48; SCAI E vs. SCAI D: RR = 1.03; 95% CI 0.85–1.31, *P* < 0.0001; *Figure* 2A). Between SCAI D and SCAI E patients, only minimal differences were observed with a numerically higher mortality rate in SCAI D patients. As detailed in the Discussion section, this likely reflects the post‐hoc definition of SCAI stages: the SCAI E group was defined solely by baseline lactate levels, including patients with either rising or falling values, whereas the SCAI D group comprised only those with lactate levels above baseline or death within 8 h. In total, 32 out of the 417 patients (7.7%) died before 8 h, including 1/215 patients (0.5%) in the SCAI C group, 13/56 (23.2%) in the SCAI D group and 18/146 patients (12.3%) in the SCAI E group. The patient dying before 8 h in the SCAI C group is a patient with missing lactate values, who was classified in the SCAI C group for the main ECLS‐SHOCK publication. Exclusion of this one patient did not affect the main results of this analysis. The mortality rates of SCAI D and SCAI E patients are similar (SCAI D vs. SCAI C: RR = 1.80; 95% CI 1.31–2.48; SCAI E vs. SCAI C: RR = 1.84; 95% CI 1.45–2.34; SCAI E vs. SCAI D: RR = 0.98; 95% CI 0.73–1.31, *P* < 0.0001; *Figure* 2B). In line with this, rates of renal replacement therapy (C vs. D vs. E: 7.0% vs. 19.6% vs. 13.7%, *P* = 0.031) and poor neurological outcomes at 30 days (CPC 3–5; C vs. D vs. E: 17.2% vs. 44.4% vs. 36.5%, *P* < 0.001) were higher in SCAI Stages D and E. Notably, higher SCAI stages were associated with a significantly higher incidence of safety endpoints, such as stroke and/or systemic embolization (C vs. D vs. E: 1.4% vs. 3.6% vs. 6.2%, *P* = 0.014) and moderate or severe bleeding (C vs. D vs. E: 12.1% vs. 23.2% vs. 20.5%, *P* = 0.026).

### ECLS versus conservative treatment comparing different SCAI stages

**Figure 3:**
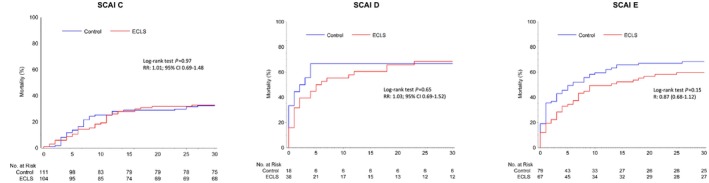
Kaplan–Meier analysis showing the effectivity of ECLS compared with no ECLS with regard to 30 day all‐cause mortality stratified by SCAI stage. SCAI C: log‐rank *P* = 0.97; SCAI D: log‐rank *P* = 0.65; SCAI E: log‐rank *P* = 0.15. CI, confidence interval; ECLS, extracorporeal life support; RR, relative risk; SCAI, Society for Cardiovascular Angiography and Interventions.

*Figure* 1 depicts the number of patients in each SCAI stage randomized to ECLS or control. Notably, no significant interaction was observed between SCAI stage at admission and the treatment effect of ECLS regarding the primary endpoint 30 day all‐cause mortality, even though a numerical difference in favour of ECLS was observed in the SCAI E group (ECLS vs. control SCAI C: 32.7% vs. 32.4%; SCAI D: 68.4% vs. 66.7%; SCAI E: 59.7% vs. 68.4%, *P* for interaction = 0.65), *Figure* 3.

In the group of SCAI C patients, ECLS treatment was associated with increased bleeding events (ECLS vs. control: 26.0% vs. 9.0%, *P* < 0.001) and peripheral ischaemic vascular complications (ECLS vs. control: 10.6% vs. 0.9%, *P* = 0.002). In SCAI D patients, only bleeding events (ECLS vs. control: 36.8% vs. 5.6%, *P* = 0.014) were increased by ECLS treatment. Contrarily, no significant differences regarding safety events were observed in SCAI E patients who had a higher baseline risk (*Table* 2).

**Table 2:** Endpoints at ≤30 days comparing ECLS versus control according to SCAI stage

	SCAI C			SCAI D			SCAI E		
	ELCS	Control	*P*	ELCS	Control	*P*	ELCS	Control	*P*
Number of patients	104 (48.4%)	111 (51.6%)		38 (67.9%)	18 (32.1%)		67 (45.9%)	79 (54.1%)	
Primary endpoint									
Death at 30 days	32.7% (34/104)	32.4% (36/111)	0.97	68.4% (26/38)	66.7% (12/18)	0.90	59.7% (40/67)	68.4% (54/79)	0.28
Secondary endpoints at 30 days									
Renal replacement therapy	4.8% (5/104)	9.0% (10/111)	0.23	15.8% (6/38)	27.8% (5/18)	0.29	9.0% (6/67)	17.7% (14/79)	0.12
Repeat revascularization (PCI and/or CABG)	8.7% (9/104)	10.8% (12/111)	0.59	10.5% (4/38)	5.6% (1/18)	0.54	7.5% (5/67)	11.4% (9/79)	0.42
Poor neurological outcome (CPC 3–5)	17.1% (12/70)	17.3% (13/75)	0.98	50.0% (6/12)	33.3% (2/6)	0.50	37.0% (10/27)	36.0% (9/25)	0.94
Safety endpoints									
Peripheral ischaemic vascular complication	10.6% (11/104)	0.9% (1/111)	0.002	10.5% (4/38)	11.1% (2/18)	0.95	11.9% (8/67)	6.3% (5/79)	0.24
Stroke	1.0% (1/104)	1.8% (2/111)	0.60	5.3% (2/38)	0.0% (0/18)	0.32	7.5% (5/67)	3.8% (3/79)	0.33
All bleeding	26.0% (27/104)	9.0% (10/111)	<0.001	36.8% (14/38)	5.6% (1/18)	0.014	26.9% (18/67)	20.3% (16/79)	0.35
Moderate or severe bleeding (BARC ≥ 3)	19.2% (20/104)	5.4% (6/111)	0.002	31.6% (12/38)	5.6% (1/18)	0.031	25.4% (17/67)	16.5% (13/79)	0.18

*Note*: Displayed are percentages and numbers. *P* values: Pearson *χ* ^2^ test.

Abbreviations: BARC, Bleeding Academic Research Consortium; CABG, coronary artery bypass grafting; CPC, cerebral performance category; ECLS, extracorporeal life support; *N*, number of observations; PCI, percutaneous coronary intervention; SCAI, Society for Cardiovascular Angiography and Interventions.

Notably, due to small numbers in the SCAI D and E groups, the statistical power of this analysis is limited.

## Discussion

The present study investigated the prognostic impact of the SCAI stages at admission and compared the treatment effect and safety of ECLS according to different SCAI stages in 417 patients with AMI‐CS enrolled in the ECLS‐SHOCK trial. The results confirm the prognostic value of admission SCAI stages in patients with AMI‐CS and planned early revascularization, with a lower risk of 30 day mortality in patients with SCAI shock Stage C, who accounted for the majority enrolled in ECLS‐SHOCK. Furthermore, higher SCAI stages were associated with an increased incidence of adverse events. Notably, ECLS did not reduce the risk of all‐cause mortality at 30 days, irrespective of SCAI stage at admission.

The value of the SCAI shock classification for prediction of mortality in patients with AMI‐CS has been shown in several retrospective studies.^10^, ^11^, ^12^, ^13^, ^14^, ^15^, ^16^, ^17^, ^18^, ^19^, ^20^ The results of this retrospective subanalysis show that this is also the case for the patients enrolled in the ECLS‐SHOCK trial. Mortality rates observed in the present analysis are in line with those described in prior validation analyses of the SCAI classification.^10^, ^12^, ^13^, ^14^, ^15^, ^16^, ^17^, ^18^, ^19^, ^20^ However, we observed only a minimal difference in mortality rates between SCAI D and E patients and even a numerical increase in mortality in SCAI D compared with SCAI E patients, which differs from the majority of prior studies. This is also reflected in some of the baseline characteristics, such as the use of vasoactive drugs and might be partly explained by the low numbers of patients (*n* = 54) included in the SCAI D group. The most likely explanation might be the post‐hoc definition of SCAI stages used in the current analysis. Patients assigned to the SCAI E group needed to have higher baseline lactate levels compared with the SCAI D group (SCAI E: >8 mmol/L, SCAI D: 3–8 mmol/L). However, while the SCAI E group was solely defined by baseline lactate levels, thus including patients with both increasing and decreasing lactate levels at follow‐up, the SCAI D group included only patients with a higher‐than‐baseline lactate level or death at 8 h, increasing the odds for inclusion of early deaths. Nevertheless, other baseline characteristics reflecting shock severity, such as the incidence of oliguria and serum creatinine levels, showed a stepwise increase between SCAI Stage C, D and E, as reported in the other validation studies. To further investigate this issue, we performed a sensitivity analysis excluding patients who died within 8 h after admission. Herein, the difference between SCAI D and SCAI E patients was no longer detectable despite the SCAI D group having the highest proportion of early deaths. Regardless, this analysis underlines the finding that severely elevated or rising serum lactate levels during the early phase of AMI‐CS therapy portend a substantially higher mortality.^22^ Furthermore, our data emphasize that a gradient in risk exists across the SCAI shock classification among patients receiving ECLS, such that simply receiving ECLS should not be used as a sole criterion for categorizing the patient in SCAI shock Stage E.

The observed increase in safety events in higher SCAI stages is likely due to a higher degree of critical illness, leading to a stronger inflammatory response, to derangements of the coagulation system and to a higher incidence of organ failure with increasing shock severity. Consequently, patients in higher SCAI stages are more likely to present with pronounced hemodynamic instability or impending collapse, so that medical measures need to be taken even more urgently, which in turn might increase complication rates. The number of patients who had undergone CPR before randomization and also the duration of CPR differed numerically between groups showing the highest rates and longest duration of CPR in the SCAI E group. However, this difference did not reach statistical significance. Unfortunately, an analysis of the efficacy of ECLS according to SCAI stages excluding resuscitated patients is not feasible due to the predominance of resuscitated patients enrolled in this trial. However, another predefined ECLS‐SHOCK subanalysis will investigate the prognostic influence of resuscitated cardiac arrest on the efficacy and safety of ECLS.^23^

ECLS was associated with a higher rate of adverse events overall, particularly in the SCAI Stage C group who had net harm with ECLS. No statistical interaction was observed for the treatment effect of ECLS according to different SCAI stages, arguing against shock severity as a modifier of the effect of ECLS on mortality. However, crude mortality was numerically lower for SCAI Stage E patients who received ECLS, raising the possibility that the sickest patients might benefit from ECLS treatment. Taken together, independently of the SCAI stages, the available data are not sufficient to characterize the subgroup of patients who might benefit from ECLS therapy. As stated in the 2025 ACC/AHA/ACEP/NAEMSP/SCAI guidelines for the management of patients with acute coronary syndromes, routine use of ECLS therapy is not recommended (class of recommendation 3).^24^ Therefore, the decision of whether or not to apply ECLS therapy should be taken individually by a multidisciplinary shock team.

Several limitations need to be acknowledged for the present study. Most importantly, by replicating the classification used in the original ECLS‐SHOCK publication,^3^ the SCAI shock stages were defined post hoc based on lactate levels. As explained above in more detail, SCAI D was defined as a lactate level of >3–8 mmol/L at baseline with a higher‐than‐baseline lactate level at 8 h or death within 8 h, which led to the inclusion of a high number of early deaths in the SCAI D group and a numerically higher mortality rate compared with SCAI E patients. Various clinical findings have been used in combination with lactate levels to define the SCAI shock stages in other studies, and it is possible that using a broader array of variables could have refined our SCAI shock classification. We specifically did not have data on vasopressor and inotrope doses, which have been used to define the SCAI stages in some prior studies. In contrast, other SCAI shock stages validation studies also used subjective parameters like MCS use or grouping of parameters, which are well known to increase mortality leaving some subjectivity of these validation studies. The trial was not powered for this subgroup analysis, and the modest number of patients in the SCAI D and E groups limited statistical power of this post hoc analysis to detect a treatment effect of ECLS. Furthermore, no information on long‐term all‐cause mortality was available for the present study. Finally, the ECLS‐SHOCK trial enrolled a selected cohort of patients with AMI‐CS in two European countries and had specific inclusion and exclusion criteria resulting in enrolment of less than half of screened patients. Accordingly, this study population is not an all‐comers AMI‐CS cohort, and these findings may not apply to all patients with AMI‐CS or to CS not caused by AMI.

## Conclusions

In conclusion, in patients with AMI‐CS included in the ECLS‐SHOCK trial, SCAI stages at admission were predictive for mortality and for the incidence of safety events. However, the effect of ECLS treatment on mortality was not affected by the SCAI stage, and patients who received ECLS had a higher rate of safety events. This analysis does not support the use of the SCAI shock classification, as defined in our study, as a stand‐alone metric to determine which patients with AMI‐CS should receive ECLS.

## Funding

Supported by the Else Kröner Fresenius Foundation, the German Heart Research Foundation, and the Helios Health Institute (formerly Leipzig Heart Institute).

## Conflict of interest statement

The authors declare that they do not have any conflict of interest.

## Supporting information


**Table S1.** Other mechanical circulatory support in patients without ECLS — no./total no. (%).
