# Supplementary material for: Prognostic impact of SCAI shock severity classes in AMI‐related cardiogenic shock: A sub‐study of the ECLS‐SHOCK Trial
Source: ESC Heart Fail. 2025 Oct 13;12(6):4359–68. doi: 10.1002/ehf2.15446 (PMC12719867; doi:10.1002/ehf2.15446)
Supplement: Supplementary file 1 — Table S1. Other mechanical circulatory support in patients without ECLS — no./total no. (%). [file EHF2-12-4359-s001.docx]

| **Table S1. Other mechanical circulatory support in patients without ECLS — no./total no. (%)** | | | |  |
| --- | --- | --- | --- | --- |
|  | **SCAI C** | **SCAI D** | **SCAI E** | **P-value** |
|  | | | |  |
|  | | | |  |
| **Number of patients** | 10/111 (9.0%) | 5/18 (27.8%) | 13/79 (16.5%) | 0.12 |
| **IABP (N)** | 0 | 1 | 0 |  |
| **Impella 2.5 (N)** | 0 | 0 | 1 |  |
| **Impella CP (N)** | 10 | 4 | 10 |  |
| **Impella 5.0 (N)** | 0 | 0 | 1 |  |
| **Other (N)** | 0 | 0 | 2 |  |
| IABP: Intraaortic Balloon Pump | | | | |

Table S1: Other mechanical circulatory support used in patients without ECLS
